# Hypoxia and the Hypoxic Response Pathway Protect against Pore-Forming Toxins in *C. elegans*

**DOI:** 10.1371/journal.ppat.1000689

**Published:** 2009-12-11

**Authors:** Audrey Bellier, Chang-Shi Chen, Cheng-Yuan Kao, Hediye N. Cinar, Raffi V. Aroian

**Affiliations:** 1 Section of Cell and Developmental Biology, University of California, San Diego, La Jolla, California, United States of America; 2 United States Food and Drug Administration, Center for Food Safety and Applied Nutrition, Division of Virulence Assessment, Laurel, Maryland, United States of America; University of Illinois, United States of America

## Abstract

Pore-forming toxins (PFTs) are by far the most abundant bacterial protein toxins and are important for the virulence of many important pathogens. As such, cellular responses to PFTs critically modulate host-pathogen interactions. Although many cellular responses to PFTs have been recorded, little is understood about their relevance to pathological or defensive outcomes. To shed light on this important question, we have turned to the only genetic system for studying PFT-host interactions—*Caenorhabditis elegans* intoxication by Crystal (Cry) protein PFTs. We mutagenized and screened for *C. elegans* mutants resistant to a Cry PFT and recovered one mutant. Complementation, sequencing, transgenic rescue, and RNA interference data demonstrate that this mutant eliminates a gene normally involved in repression of the hypoxia (low oxygen response) pathway. We find that up-regulation of the *C. elegans* hypoxia pathway via the inactivation of three different genes that normally repress the pathway results in animals resistant to Cry PFTs. Conversely, mutation in the central activator of the hypoxia response, HIF-1, suppresses this resistance and can result in animals defective in PFT defenses. These results extend to a PFT that attacks mammals since up-regulation of the hypoxia pathway confers resistance to *Vibrio cholerae* cytolysin (VCC), whereas down-regulation confers hypersusceptibility. The hypoxia PFT defense pathway acts cell autonomously to protect the cells directly under attack and is different from other hypoxia pathway stress responses. Two of the downstream effectors of this pathway include the nuclear receptor *nhr-57* and the unfolded protein response. In addition, the hypoxia pathway itself is induced by PFT, and low oxygen is protective against PFT intoxication. These results demonstrate that hypoxia and induction of the hypoxia response protect cells against PFTs, and that the cellular environment can be modulated via the hypoxia pathway to protect against the most prevalent class of weapons used by pathogenic bacteria.

## Author Summary

Bacteria make many different protein toxins to attack our cells and immune system in order to infect. Amongst them, pore-forming toxins (PFTs), which punch holes in the protective plasma membrane that surrounds cells, are by far the most abundant and constitute important virulence factors. Since the integrity of the plasma membrane is fundamental to maintaining the normal intracellular environment, the breaching of the plasma membrane by PFTs results in many and dramatic intracellular responses. However, we know little about the relevance of these responses to cell survival or cell intoxication. Here, using the only genetic system for studying pore-forming toxin effects in a whole animal, we show that the same response that protects cells against low oxygen stress unexpectedly also protects cells against pore-forming toxins. Mutations in the animal that hyper-activate the low oxygen response actually make animals resistant to pore-forming toxin attack, whereas mutations that inactivate the low oxygen response make animals more susceptible. Furthermore, a low oxygen environment itself is protective against pore-forming toxins. These data show a new and powerful connection between low oxygen responses and defense against the single most common mode of bacterial attack.

## Introduction

Pore-forming toxins (PFTs) are by far the most abundant and amongst the most important bacterial protein virulence factors [1]. These toxins, secreted by many pathogenic bacteria, function by binding to receptors on host cell plasma membrane, oligomerizing, inserting, and then forming holes [2]. The importance of PFTs in promoting bacterial pathogenesis has been demonstrated by numerous experiments where individual PFTs have been genetically deleted from pathogenic bacteria and the bacteria then tested for reduced virulence [3]. Examples of PFTs that contribute significantly to bacterial virulence include α-toxin by *Clostridium septicum*, streptolysins by Group A and B Streptococci, α-toxin by *Staphylococcus aureus*, *Vibrio cholerae* cytolysin (VCC), α-hemolysin from uropathogenic *E. coli*, cytolysin from *Enterococcus faecalis*, and crystal (Cry) proteins from *Bacillus thuringiensis* (Bt).

Although they are expressed by many bacterial pathogens and are broadly important as potentiators of infection, the effects of these toxins on host cells have been vastly understudied. There are several reasons for this lack of attention. First, their mechanism of action is deceptively simple. Second, most of the attention has been given to understanding how PFTs can change conformation from secreted, soluble proteins to insoluble proteins embedded in the plasma membrane. Third, because breaching of the plasma membrane is a major insult to a cell, a multitude of cellular responses to PFTs have been reported, including Ca^2+^ influx, K^+^ efflux, increased endocytosis/exocytosis, vacuolization, necrosis, and apoptosis [3],[4],[5],[6]. Because the responses are so large and extensive, it has been daunting to determine whether these responses contribute to defense, intoxication, both, or neither. Fourth, most of the studies carried out to date involved cells in isolated culture, which does not always accurately recreate the response of cells to toxins in the context of intact tissue.

To address many of these limitations, an excellent genetic system for studying PFT responses in an intact animal has recently emerged: the *Bacillus thuringiensis* (Bt) Crystal (Cry) PFT – *Caenorhabditis elegans* toxin-host interaction system [7]. *C. elegans* has become an important genetically tractable organism for studying immune responses to bacterial pathogens [8]. Bt is thought to be a natural pathogen of *C. elegans* [9],[10],[11] and is famous for the production of three-domain PFTs that are widely used in insect biocontrol [12]. The interaction of Cry proteins with *C. elegans* allowed for the first molecular PFT defense pathway identified, p38 mitogen-activated protein kinase (MAPK) pathway [13]. Loss of the p38 MAPK pathway was shown to result in loss of protection against Cry PFTs in *C. elegans* and was subsequently shown to result in loss of protection against PFTs in mammalian cells as well [13],[14]. This same system was used to discover that the unfolded protein response (UPR) is also required for PFT defenses as a downstream target of the p38 MAPK pathway [15]. Both the p38 MAPK pathway and the UPR were demonstrated to be activated by PFTs in *C. elegans* and mammals [15],[16]. Apart from these studies, only one other study to date has demonstrated a specific molecular pathway as involved in PFT responses [17].

Since, when studying intracellular PFT response pathways in the past, we have screened for *C. elegans* mutants hypersensitive to PFTs [13],[15], we reasoned that we could learn something different by screening for the opposite phenotype– *C. elegans* mutants resistant to PFTs. The reason for this assumption is that no intracellular pathway mutants were known that can make cells resistant to PFTs in general. Here we report on the results of a PFT resistance screen and find, unexpectedly, that resistance can be achieved by mutations that up-regulate the *C. elegans* low oxygen (hypoxia) response. Elimination of HIF-1 (hypoxia inducible factor 1), the main effector of the hypoxia pathway, abrogates this resistance and can lead to PFT hypersensitivity. This protection applies to multiple different PFTs and is clearly distinguished from the role of the HIF-1 pathway in other stress responses and aging. Furthermore, the hypoxia pathway is activated in response to PFTs, and low oxygen is itself protective against PFT attack. Our results indicate that the hypoxia/low oxygen response is likely to be of general importance for cellular responses to small-pore PFTs.

## Results

### Isolation and identification of a mutant resistant to Bt Cry21A toxin

To identify pathways important for cellular responses to PFTs, we screened for mutants resistant to the PFT Cry protein, Cry21A. Cry21A is a three-domain Cry protein that targets nematodes and is in the same family as Cry5B [11]. Like Cry5B [18], secondary structure programs predict Cry21A contains all the α helical segments that are involved in pore-formation in three-domain Cry proteins [19]. All three-domain Cry proteins, like Cry5B and Cry21A, are believed to act as PFTs, and pore-forming activity has been demonstrated for all Cry proteins so studied to date [12]. In the past, we have screened for mutants resistant to Cry5B, which has given rise to detailed understanding of the Cry5B receptor [20],[21],[22] but not to information on intracellular responses to Cry PFTs. Our rationale for screening for Cry21A PFT resistant animals was that it could elucidate new information about how cells respond to PFTs since Cry5B resistant mutants are only weakly resistant to Cry21A.

**Figure 1:**
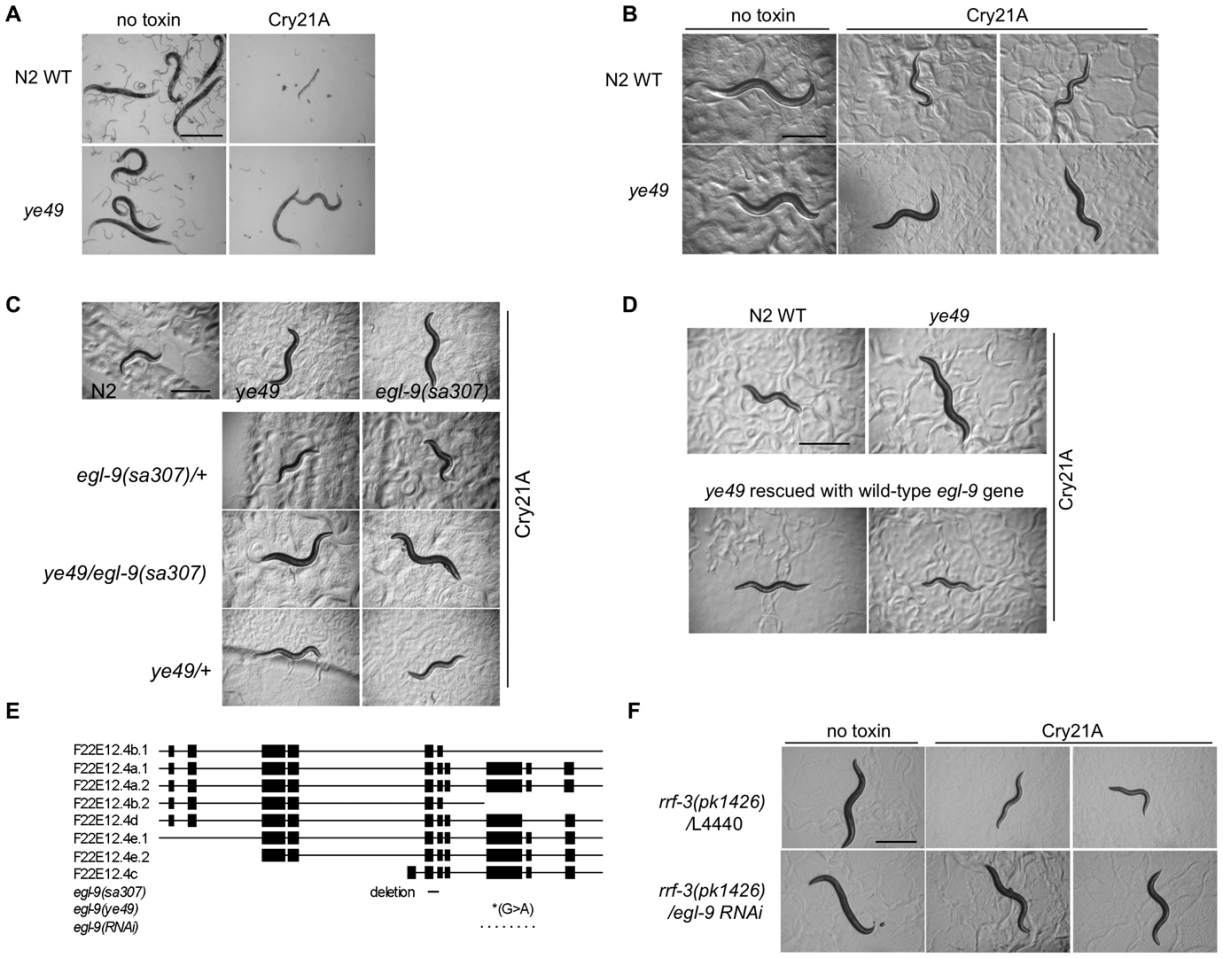
Cry21A PFT-resistant *ye49* mutates *egl-9*. Hermaphrodites from N2 wild-type and *ye49* animals after feeding on (A) Bt spore crystal lysates for 72 h without or with Cry21A crystal protein or (B) *E. coli* expressing no toxin or Cry21A. *ye49* animals on Cry21A from either Bt or *E. coli* are clearly healthier than wild-type animals on Cry21A, as they are bigger, darker, and more motile. (C) Complementation tests. Images of representative animals 48 h after feeding on *E. coli* expressing Cry21A. Top row: controls showing relative sickness of wild-type N2 animals on Cry21A compared to *ye49* and *egl-9(sa307)* animals on Cry21A. Bottom rows: heterozygous over wild type controls showing *egl-9(sa307)/+* and *ye49/+* animals are sensitive to Cry21A; *ye49/egl-9(sa307)* animals showing resistance to Cry21A. These animals are also all heterozygous for *dpy-17(e164)*, used as a marker to distinguish self from cross progeny. (D) Rescue experiments. Images of representative animals 48 h after feeding on *E. coli* expressing Cry21A. Top: controls showing relative health of wild-type N2 and *ye49* animals on Cry21A. Bottom: animals of the genotype *ye49* transformed with genomic wild-type *egl-9* DNA showing that expression of *egl-9* in *ye49* animals rescues Cry21A resistance. (E) Location of amber mutation in *egl-9* gene associated with *ye49* (*) as well as locations of the *egl-9(sa307)* deletion (bar) and *egl-9(RNAi)* clone (dotted line) from the Ahringer library [59]. Boxes represent exons. (F) RNAi of *egl-9* results in animals resistant to Cry21A. L4440 is empty vector RNAi control (no gene knock down). RNAi animals were fed Cry21A for 24 hours. Scale bar is 0.5 mm in this and all figures unless otherwise specified.

To perform this screen, *C. elegans* hermaphrodites were mutagenized with EMS and allowed to self-fertilize for two generations. Sixty eight thousand F2 mutagenized hermaphrodites were fed an intoxicating dose Cry21A PFT and then screened for those that resisted intoxication. One mutant line, allele *ye49*, was identified that bred true and is resistant to Cry21A PFT produced either from Bt or *E. coli* (Figures 1A and 1B).

To identify the gene mutated in *ye49*, we performed standard three-factor and single-nucleotide mapping experiments, which narrowed the search to a region on chromosome V, between markers snp_F15H10 and snp_T21C9, that includes 43 genes. Mutation in one of the genes in this region, *egl-9*, had been previously identified as resistant to cyanide produced by *Pseudomonas aeruginosa* PA01 [23]. We therefore performed complementation testing between *ye49* and the *egl-9* null allele *egl-9(sa307)* and found that *ye49/egl-9(sa307)* animals are resistant to Cry21A PFT, indicating *ye49* fails to complement *egl-9(sa307)* and most probably mutates the same gene (Figure 1C). Furthermore, injection of an extrachromosomal array bearing the *egl-9* promoter and coding region restored wild-type Cry21A susceptibility to *ye49* animals (Figure 1D). In addition, sequencing of *egl-9* cDNA isolated from the *ye49* mutant identified a point mutation (W_508_-to-stop) that upon translation is predicted to truncate the protein in the prolyl hydroxylase domain, thereby eliminating protein hydroxylase function (Figure 1E). These results demonstrate that Cry21A PFT resistance phenotype associated with *ye49* is due to loss of *egl-9* function mutation. As predicted from this conclusion, feeding of double-stranded RNA to cause RNA interference (RNAi) results in animals resistant to Cry21A (Figure 1F).

### The hypoxia pathway is required for protection against Cry PFTs

**Figure 2:**
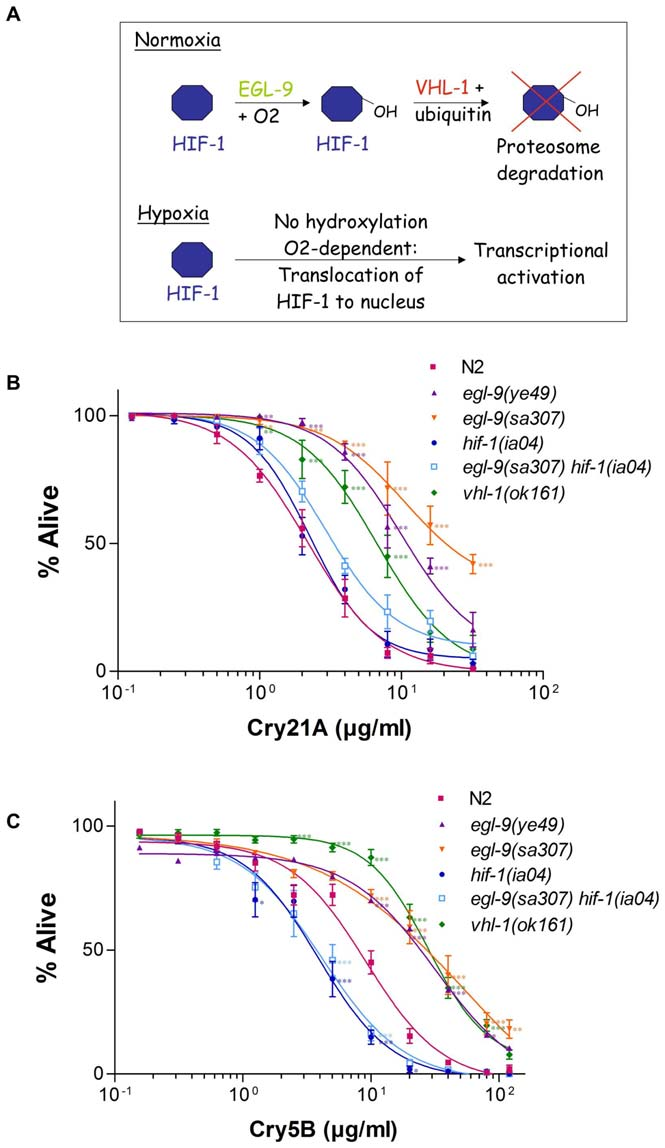
Quantitative response of *egl-9* and HIF-1 pathway mutants to Cry PFTs. (A) Schematic illustrating O_2_-dependent regulation of HIF-1 activity. The O_2_-dependent prolyl hydroxylation of HIF-1 by EGL-9/PHD increases its affinity to VHL-1, leading to ubiquitylation and destruction. (B, C) Dose-dependent mortality assays were performed using (B) Cry21A spore crystal lysates or (C) purified Cry5B to quantitatively compare sensitivities of wild-type N2, *egl-9* mutants, and HIF-1 pathway mutants to PFTs. Each data point shows the mean and standard errors of the mean of results from three independent experiments (three wells per experiment; on average, 180 animals per data point). Statistical differences between mutant strains and N2 are given for each concentration using P values represented by asterisks as follows: * P<0.05; ** P<0.01; *** P<0.001. Percent alive at specific doses and LC_50_ values are reported in Table 1.

The EGL-9 protein is a prolyl hydroxylase and functions in the *C. elegans* low oxygen response (hypoxia) pathway (Figure 2A; [24]). The ability of cells to respond to hypoxia is mediated by a transcription factor called HIF-1α. Under normal oxygen (normoxia) conditions, HIF-1α (called HIF-1 in *C. elegans*) is hydroxylated by a prolyl hydroxylase (EGL-9 in *C. elegans* or PHD in mammals) that then increases HIF-1's affinity for von Hippel-Lindau tumor suppressor protein (called VHL-1 in *C. elegans*), part of an E3 ubiquitin ligase complex. Association of HIF-1 with VHL-1 eventually leads to HIF proteasomal degradation. When EGL-9 is disabled by mutation, HIF-1 is stabilized at constitutively high levels even under normoxic (normal oxygen) conditions [25].

Since loss of EGL-9 function confers resistance to Cry21A PFT, we hypothesized that other elements of the hypoxia pathway might be important as well. We therefore performed quantitative dose-dependent mortality assays using null or putative null alleles of all the above elements of the hypoxia pathway. L4 staged animals from each genotype and wild-type N2 were placed in numerous doses of Cry21A PFT or Cry5B PFT and scored for viability after a few days (Figures 2B and 2C).

As predicted from the above studies, we find that animals lacking EGL-9 are quantitatively resistant to Cry PFTs. At doses from 1–16 µg/mL Cry21A and 10–80 µg/mL Cry5B PFTs, *egl-9(sa307)* and *egl-9(ye49)* animals are resistant to PFT-induced mortality relative to wild-type animals, with resistance strongest at higher PFT doses (Figures 2B and 2C; Table 1). For example, 7×–10× more *egl-9* mutant animals are alive at 8 µg/mL Cry21A or 40 µg/mL Cry5B PFT than wild-type animals at the same doses (P<0.001; Table 1; note, direct dose comparison between Cry21A and Cry5B toxicity is not possible since Cry21A assays are performed with Bt spore-crystal lysates and Cry5B assays with purified protein). Based on LC_50_ values (concentration at which 50% of the animals are dead), loss of EGL-9 results in 3–5 fold resistance over wild-type animals to Cry21A or Cry5B PFTs (Table 1). Note, since all our mortality assays are carried out in liquid medium, resistance to the PFT cannot be attributed to improved avoidance behaviors. Cry21A PFT resistance was also confirmed using a quantitative brood size assay (Figure S1).

**Table 1:** Data and analyses for Cry21A and Cry5B dose-dependent mortality assays.

	Cry21A concentration (µg/mL)				
Strains	1 %alive	2 %alive	4 %alive	8 %alive	16%alive
N2	76.50	55.96	28.59	7.21	5.96
*egl-9(ye49)*	100**	97.49***	85.81***	56.65***	41.14***
*egl-9(sa307)*	97.86**	95.76***	89.18***	71.52***	57.10***
*hif-1(ia04)*	91.13	52.90	32.03	10.60	8.51
*egl-9(sa307)hif-1(ia04)*	89.99	70.31	41.22	23.26*	19.54
*vhl-1(ok161)*	99.38**	82.86***	72.10***	44.96***	15.24
N2	76.19	44.32	18.63	3.60	0.69
*rhy-1(ok1402)*	95.47*	85.82***	77.01***	63.35***	40.51***

	Cry5B concentration (µg/mL)				
Strains	5 %alive	10 %alive	20 %alive	40 %alive	80 %alive
N2	72.22	45	15.32	4.64	0.65
*egl-9(ye49)*	79.73	70.04***	58.64***	33.88***	15.78*
*egl-9(sa307)*	79.93	71.74***	59.22***	39.85***	20.23***
*hif-1(ia04)*	38.29***	14.85***	1.88*	1.11	1.05
*egl-9(sa307)hif-1(ia04)*	45.87***	16.32***	4.26	1.73	0.69
*vhl-1(ok161)*	91.36***	87.31***	63.16***	34.70***	19.55***

Cry21A assay	LC_50_ (µg/mL)	Standard deviation	Relative resistance (LC_50_ wild type/LC5_50_ mutant)
N2	4.8	2.1	
*egl-9(ye49)*	17.1**	3.5	3.5
*egl-9(sa307)*	25.0***	2.9	5.2
*hif-1(ia04)*	5.5	1.9	1.1
*egl-9(sa307)hif-1(ia04)*	8.7^a^^,^ b	2.8	1.8
*vhl-1(ok161)*	11.6	3.3	2.4
N2	3.1	0.9	
*rhy-1(ok1402)*	17.4*	3.9	5.7
**Cry5B assay**			
N2	7.1	1.3	
*egl-9(ye49)*	22.8	7.0	3.2
*egl-9(sa307)*	27.9**	15.8	3.9
*hif-1(ia04)*	3.4	1.4	0.5
*egl-9(sa307)hif-1(ia04)*	3.7^c^^,^ b	1.6	0.5
*vhl-1(ok161)*	28.7**	8.0	4.0

Statistical differences between mutant strains and N2 wild-type are given for each concentration and LC_50_ using P values represented by asterisks as follow: * P<0.05, ** P<0.01, *** P<0.001.

a P value<0.001 for comparison with *egl-9(sa307)*.

b P value>0.05 for comparison with *hif-1(ia04)*.

c P value<0.01 for comparison with *egl-9(sa307)*.

We also found that *vhl-1(ok161)* mutant animals are resistant over a similarly wide range of Cry21A and Cry5B PFT doses (Figures 2B and 2C; Table 1). For example, 6.2× and 7.4× more *vhl-1* mutant animals are alive at 8 µg/mL Cry21A and 40 µg/mL Cry5B, respectively, than wild-type animals. Based on LC_50_ values, *vhl*-*1* mutant animals are 4× resistant to Cry5B PFT. We also tested *rhy-1(ok161)* mutant animals on Cry21A PFT. RHY-1 (*r*egulator of *hy*poxia-inducible factor) antagonizes HIF-1 function by inhibiting expression of some HIF-1 target genes via a VHL-1 independent pathway [26]. Animals lacking RHY-1 are also resistant to Cry21A (Table 1; Figure S2). Based on LC_50_ values, animals lacking RHY-1 are 5.7× resistant to Cry21A PFT (Table 1). These data demonstrate that loss of function mutations in genes that normally antagonize HIF-1 function all result in resistance to Cry protein PFTs. In other words, stimulation of HIF-1 function via removal of HIF-1 inhibitory factors results in PFT resistance.

To confirm that the resistance associated with *egl-9* mutants was going through HIF-1, we looked at the dose-dependent response of *hif-1(ia04)* and *egl-9(sa307) hif-1(ia04)* double mutant animals to Cry21A and Cry5B PFTs. When fed Cry21A, *hif-1(ia04)* animals have a response that is indistinguishable from wild-type animals (Figure 2B; Table 1). *egl-9(sa307) hif-1(ia04)* double mutant animals have a statistically identical response to Cry21A as wild-type animals at all doses except at 8 µg/mL (Table 1). Furthermore the LC_50_ values of *hif-1(ia04)* and *egl-9(sa307) hif-1(ia04)* animals on Cry21A PFT are statistically identical (P>0.05) but both statistically different than that of *egl-9(sa307)* (P<0.01; Table 1). These results have been qualitatively confirmed using RNAi—whereas wild-type animals subject to *egl-9* RNAi are resistant to Cry21A, *hif-1(ia04)* mutant animals subject to *egl-9* RNAi are not (Figure S3). Similarly, whereas *egl-9(sa307)* mutants are resistant to Cry21A, this resistance is suppressed by RNAi of *hif-1*. Thus, HIF-1 is required for Cry21A resistance mediated by loss of EGL-9.

The results with Cry5B PFT are similar to those of Cry21A (Figure 2C; Table 1) in that HIF-1 is required for Cry5B resistance mediated by loss of EGL-9 (*i.e.*, loss of HIF-1 suppresses Cry5B PFT resistance associated with loss of EGL-9). There is, however, one striking difference between the *hif-1* results with Cry21A and Cry5B. Both *hif-1(ia04)* and *egl-9(sa307) hif-1(ia04)* animals are hypersensitive to Cry5B PFT relative to wild-type animals. That is, animals lacking HIF-1 are more readily killed by Cry5B PFT than wild-type animals, especially at doses ∼5–10 µg/mL (P<0.05; Figure 2C; Table 1). Thus, *hif-1* is required for intrinsic cellular defenses (INCED) [15] against Cry5B PFT. With regards to the different results with Cry5B and Cry21A, we speculate that perhaps Cry5B PFT intoxicates more potently than Cry21A and that, whereas increased HIF-1 activity is protective against all PFTs, loss of HIF-1 activity is more acutely felt when the stronger PFT is present. In the case of Cry21A, other INCED pathways are sufficient for full protection even in the absence of HIF-1.

### The hypoxia pathway is required for defense against *V. cholerae* cytolysin

**Figure 3:**
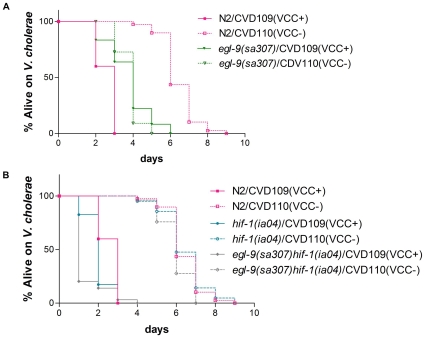
*egl-9* mutation confers resistance to *V. cholerae* VCC. (A) The survival of wild-type N2 and *egl-9(sa307)* mutant animals on *V. cholerae* CVD109(VCC+) and CVD110(VCC−) are shown. (B) The survival of wild-type N2, *hif-1(ia04)*, and *egl-9(sa307) hif-1(ia04)* mutant animals on *V. cholerae* CVD109(VCC+) and CVD110(VCC−) are shown. Quantitative data and statistical analyses for the representative experiment shown here are included in Table 2. Data for two other independent repetitions of the experiment are shown in Table S1.

Cry proteins are small-pore PFTs. To test whether or not the hypoxia pathway was more generally required for INCED against PFTs, we fed *C. elegans* two *V. cholerae* strains that differ primarily in their ability to produce another small-pore PFT, VCC. VCC is a virulence factor of *V. cholerae* and mutants lacking VCC are attenuated for pathogenesis *in vivo*, especially for strains lacking cholera toxin [27],[28]. The strains we use are CVD109(VCC+) and CVD110(VCC−) that are nearly isogenic (except for the presence of cholera toxin B subunit in CVD110, which should not matter since *C. elegans* lacks sialic acid that the B subunit binds to as part of its GM1 receptor [29]). Although both strains are pathogenic when fed to *C. elegans*, CVD109(VCC+) is more virulent than CVD110(VCC−), demonstrating that VCC is a virulence factor for *C. elegans* (Figure 3A).

Our results with hypoxia pathway mutants on CVD109(VCC+) and CVD110(VCC−) are striking and parallel those with Cry PFTs. When feeding on CVD109(VCC+), *egl-9(sa307)* animals are resistant relative to wild-type animals (Figure 3A; Table 2; median survival 4 vs. 3 days respectively; P<0.001). This resistance is dependent upon the presence of VCC since when feeding on CVD110(VCC−), *egl-9(sa307)* animals are not resistant (Figure 3A; Table 2). Similarly, *hif-1(ia04)* and *egl-9(sa307) hif-1(ia04)* animals are, as with Cry5B PFT, hypersensitive relative to wild-type animals on CVD109(VCC+) (median survival of 2, 1, and 3 days respectively; P<0.0001; Figure 3B; Table 2). This hypersensitivity is dependent upon the presence of VCC since these mutants are not hypersensitive when feeding on CVD110(VCC−) (Figure 3B; Table 2). It is interesting to note that *egl-9(sa307)* mutant animals are hypersensitive compared to wild-type animals to CVD110(VCC−) strain (median survival of 4 and 6 days respectively; P<0.0001; Figure 3A; Table 2). We speculate that while activation of the hypoxia pathway (in an *egl-9* mutant or otherwise) protects the animals against VCC and PFTs (hence *egl-9* mutants are resistant to the VCC+ strain), activation of the hypoxia pathway may make the animals more susceptible to other *V. cholerae* virulence factors. The relative contribution to these responses (protection versus susceptibility) is dependent upon which virulence factors are present and their relative contribution to virulence. In the VCC+ strain, the PFT has important function. Hence, the protective role of pathway activation can be discerned. In the VCC− strain, the PFT defense is no longer needed. Hence, the susceptible role can be discerned. It is this give-and-take interaction between the host and virulence factors that could partly explain why constitutive mutation in *egl-9* is not selected in the wild. In any event, taken together, our Cry PFT and VCC data demonstrate that stabilization of HIF-1 results in resistance to VCC PFT whereas loss of HIF-1 results in hypersensitivity to VCC PFT.

**Table 2:** Data and analyses of VCC, PA14, and lifespan assays.

*V. cholerae* assays	median survival (days)	p value for comparison with the N2 survival curves
N2/CVD109(VCC+)	3	
*egl-9(sa307)*/CVD109(VCC+)	4	<0.0001
*hif-1(ia04)*/CVD109(VCC+)	2	<0.0001
*egl-9(sa307)hif-1(ia04)*/CVD109(VCC+)	1	<0.0001
N2/CVD110(VCC−)	6	
*egl-9(sa307)*/CVD110(VCC−)	4	<0.0001
*hif-1(ia04)*/CVD110(VCC−)	6	>0.05
*egl-9(sa307)hif-1(ia04)*/CVD110(VCC−)	6	>0.05

*P. aeruginosa* PA14 assays	median survival (hours)	p value for comparison with the N2 survival curves
N2	50	
*egl-9(ye49)*	70	<0.0001
*egl-9(sa307)*	60	<0.001
N2	40	
*hif-1(ia04)*	48	<0.0001
*egl-9(sa307)hif-1(ia04)*	52	<0.0001

Lifespan analysis	median survival (days)	p value for comparison with the N2 survival curves
N2	15	
*egl-9(ye49)*	20	<0.01
*egl-9(sa307)*	17	<0.05
N2	15	
*hif-1(ia04)*	18	<0.001
*egl-9(sa307)hif-1(ia04)*	20	<0.001

### The requirement of the HIF-1 pathway for PFT response can be differentiated from other stressors

**Figure 4:**
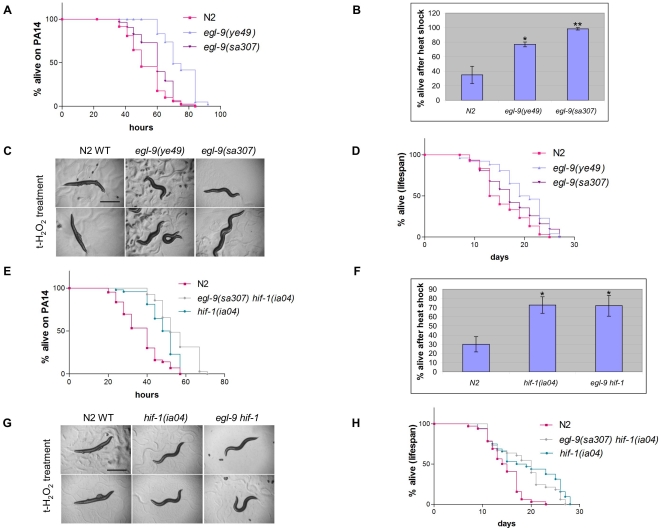
*egl-9* and *hif-1* mutant phenotypes on other stressors and on aging. (A) The survival of wild-type N2 and *egl-9* mutant animals on *P. aeruginosa* PA14 are shown for one representative assay (n>50). (B) *egl-9* mutant and N2 animals were scored for viability after 12 h exposure to 35°C. Data are averaged from three independent experiments with n>30 for each. Error bars represent standard error of the mean, * represents P values<0.05 and ** P values<0.01. (C) Comparison of *egl-9* mutant animals to wild-type animals on hydrogen peroxide. Representative worms are shown for each strain 6 h after continual treatment. Wild-type N2 animals are still alive but paralyzed, whereas *egl-9* mutant animals are active and healthy, indicative of resistance. (D) Lifespan of N2 and *egl-9* mutants feeding on *E. coli* OP50 (n>30 animals per strain). One of three representative experiments shown. (E–G) *hif-1(ia04)* and *egl-9(sa307) hif-1(ia04)* mutant animals challenged with (E) *P. aeruginosa* PA14 (one of three representative experiments shown), (F) heat shock at 35°C for 14 h, and (G) oxidative stress (hydrogen peroxide; both mutant strains are still active, unlike N2 animals). (H) Lifespan of *hif-1(ia04)* and *egl-9(sa307) hif-1(ia04)* mutant animals feeding on *E. coli* OP50. Quantitative data and statistical analyses for the experiments shown here are included in Table 2. Independent repeats for the PA14 and lifespan assays are shown in Table S1.

Because loss of EGL-9 results in resistance to PFTs (here) and cyanide [23],[30], we hypothesized that *egl-9* mutant animals might show resistance to other stressors as well. We found that, relative to wild-type animals, animals lacking EGL-9 are resistant to killing by 1) the pathogen *Pseudomonas aeruginosa* PA14; 2) heat stress; and 3) oxidative stress (Figure 4A, Table 2; Figures 4B and 4C). Since correlation between stress response and lifespan had previously been reported, such as in the *daf-2* mutant [31],[32], we tested whether loss of EGL-9 had an effect on longevity. Indeed, *egl-9(ye49)* and *egl-9(sa307)* mutant animals live longer than N2 wild-type when feeding on the standard *E. coli* strain (Figure 4D, Table 2).

To study the relationship between the hypoxia response pathway and resistance to stresses in more detail, we asked if the resistance to these different stresses via loss of EGL-9 was, as for resistance to PFTs, mediated through HIF-1. Unexpectedly, we found that *hif-1(ia04)* loss-of-function mutant animals as well as *egl-9(sa307) hif-1(ia04)* mutant animals are resistant to *P. aeruginosa* PA14 infection, heat stress, and oxidative stress (Figure 4E, Table 2; Figures 4F and 4G). Both mutant strains are also long lived (Figure 4H; Table 2). Thus, in the case of these stresses, but unlike that of PFT response, loss of either EGL-9, HIF-1, or both results in stress resistance. We speculate that, in the case of these other stresses, hydroxylation of HIF-1 by EGL-9 may result in its activation prior to degradation. Similar results have been previously reported in that mutation of either *hif-1* or *egl-9* results in *C. elegans* resistant to pathogenic *E. coli* [33]. With regards to lifespan, published studies are contradictory but there is at least one published report with *egl-9* mutants long lived and two with *hif-1* long-lived [34],[35],[36]. In any event, our results demonstrate that role of the hypoxia pathway in PFT INCED is separable from that of other stress responses.

### The hypoxia pathway functions cell-autonomously in PFT responses

**Figure 5:**
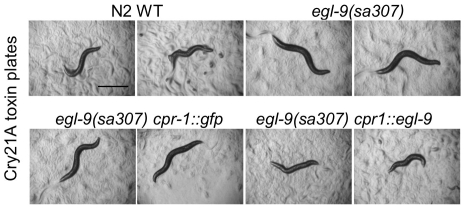
Intestinal specific expression of *egl-9* is sufficient to rescue Cry21A PFT resistance. Resistance to Cry21A was compared among wild type N2, *egl-9(sa307), egl-9(sa307)* transformed with *cpr-1::gfp*, and *egl-9(sa307)* animals transformed with *cpr1::egl-9*. Two representative worms are shown for each strain 48 h after feeding on *E. coli*-expressed Cry21A. Wild-type animals and *egl-9(sa307)* animals transformed with *cpr1::egl-9* are similarly sensitive to Cry21A whereas *egl-9(sa307)* animals and *egl-9(sa307)* animals transformed with *cpr-1::gfp* are relatively resistant.

Bt Cry PFTs attack intestinal cells [21],[22],[37]. It is possible that the hypoxia defense pathway functions within the cells targeted by the PFTs or that the hypoxia pathway is functioning cell non-autonomously. To address this question, we expressed *egl-9* under the control of various promoters including the intestinal specific *cpr-1* promoter [21],[22],[38] and the *unc-31* promoter, which is expressed in all neurons and in secretory cells of the somatic gonad [39]. We find that expression of wild-type EGL-9 under the *cpr-1* promoter in the intestinal cells of *egl-9(sa307)* animals (Figure 5), but not under the *unc-31* promoter in the neuronal or secretory cells (not shown), is sufficient to rescue the *egl-9(sa307)* Cry21A resistance phenotype. Control animals in which green-fluorescent protein (GFP) was expressed from the *cpr-1* promoter did not result in rescue. Quantitative mortality assays using two independent lines of *cpr-1::egl-9*-transformed *egl-9(sa307)* mutant animals confirm that intestinal-specific expression of EGL-9 rescues Cry21A PFT resistance to a level statistically indistinguishable from N2 wild-type (not shown). These data are consistent with the hypoxia pathway acting to directly counteract the effects of PFTs and not, for example, providing protection via altered behavior.

### The UPR and the nuclear receptor *nhr-57* are downstream effectors of the hypoxia PFT INCED

To address how the hypoxia pathway might function in protection against PFTs, we sought in two ways to find functional downstream effectors of the pathway. First, we compared known functional targets of the hypoxia pathway in *C. elegans* and asked if any of these are involved in PFT defenses. One pathway immediately surfaced, the unfolded protein response or UPR [40]. It has been recently reported that the hypoxia pathway genetically functions upstream of the XBP-1 arm of the UPR with regards to longevity in *C. elegans* [35]. Furthermore, we have previously shown that the XBP-1 is required for PFT INCED since loss of XBP-1 leads to animals that are hypersensitive to Cry5B PFT [15]. These data suggest that the XBP-1 arm of the UPR is one downstream target of the hypoxia PFT INCED.

To test this suggestion, we examined whether or not the hypoxia pathway regulates activation of the XBP-1 UPR pathway. Activation of the XBP-1 UPR pathway can readily be discerned by examining *xbp-1* mRNA, which is spliced upon activation of the pathway [41]. We indeed find that activation of the hypoxia pathway results in activation of the UPR as seen by a 1.4 fold increase in spliced *xbp-1* levels in *egl-9* mutant animals (P<0.001; see Materials and Methods). Thus, one functional downstream effector of the hypoxia pathway for PFT defenses is the XBP-1 UPR.

We conversely asked if any of the genes known to be involved in PFT INCED are known to be important for the hypoxia pathway. From over 100 PFT INCED genes we have identified in our lab, we found one and only one currently known to be regulated by the hypoxia pathway, *nhr-57*. *nhr-57* was initially identified as part of the hypoxia pathway by the fact that its expression is positively regulated by *hif-1* and negatively regulated by *egl-9* and *vhl-1* [42],[43]. In fact, *nhr-57* transcriptional activation is considered the most reliable marker for activated HIF-1 function in *C. elegans* [26]. We confirmed using quantitative PCR that in *egl-9* mutant animals, *nhr-57* transcripts are induced 15 fold and that this increase is completely dependent upon HIF-1 (data not shown). However, to date no functional role of *nhr-57* for any HIF-1-regulated pathway has been shown.

**Figure 6:**
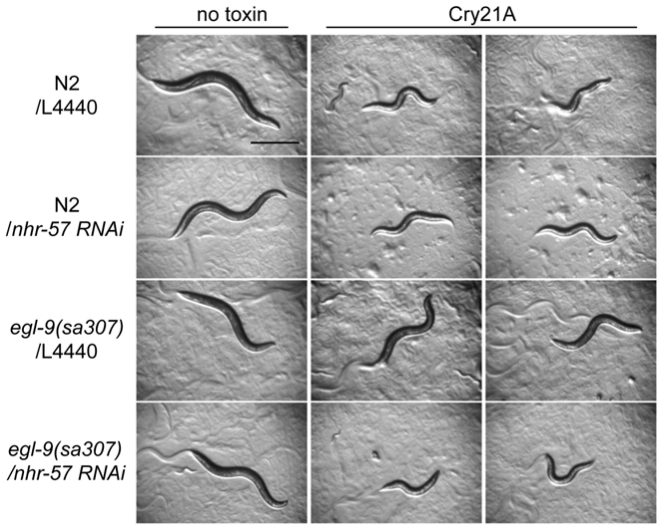
*nhr-57*, a HIF-1-target, is required for loss of *egl-9*-mediated resistance to PFT. Wild-type N2 and *egl-9(sa307)* mutant animals were treated to RNAi using either empty vector (L4440) or dsRNA for *nhr-57* and exposed to *E. coli*-expressed Cry21A PFT for 48 hours. Shown are representative animals for each condition. All animals are healthy in the absence of Cry21A. On Cry21A, whereas *egl-9(sa307)* animals are resistant (third row), *nhr-57(RNAi) egl-9(sa307)* animals are not.

We find that knock down of *nhr-57* results in animals slightly but statistically hypersensitive to Cry5B PFT (*e.g.*, 21% reduction in viability for *nhr-57* RNAi at 20 µg/mL Cry5B PFT versus vector-only RNAi control, P = 0.02; n = 90; see Materials and Methods) and therefore defective in PFT INCED. More impressively, we find that knock down of *nhr-57* completely suppresses the resistance to Cry21A PFT associated with loss of EGL-9 (Figure 6). Taken together, these results indicate that the nuclear receptor *nhr-57* is a second functional downstream effector of the hypoxia PFT defense pathway.

### The hypoxia pathway is induced by PFT and hypoxia is protective against PFT

**Figure 7:**
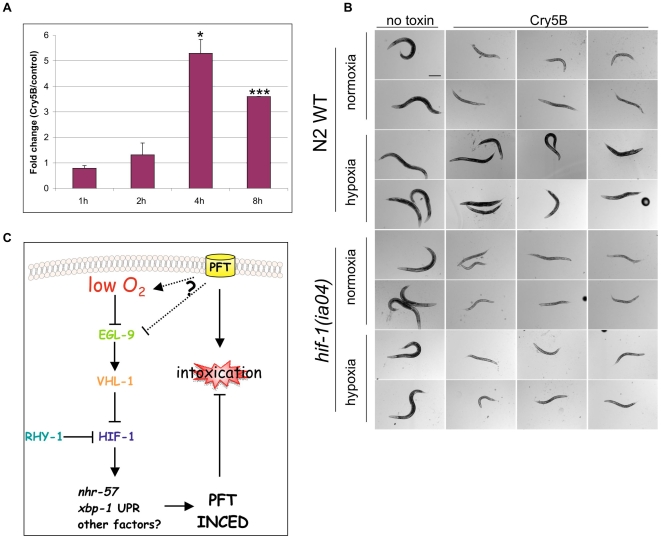
Cry5B PFT activate the hypoxia pathway and hypoxia confers protection against PFTs. (A) Quantitative real-time PCR analysis of *nhr-57* expression in *glp-4(bn2)* animals upon treatment on Cry5B for 1, 2, 4 and 8 hours relative to expression levels in no-toxin treatment animals. Data are averaged from three independent experiments. Error bars represent standard error of the mean, * represents P value<0.05 and *** P value<0.001. (B) Resistance to Cry5B PFT was compared among wild-type N2 worms in normoxia (top two rows) and in hypoxia (2% O_2_) (rows 3 and 4) and among *hif-1(ia04)* mutant animals in normoxia (rows 5 and 6) and in hypoxia (bottom two rows). Wild-type worms co-treated with hypoxia and Cry5B are significantly healthier (larger, darker color, more embryos, more motile) than worms treated with Cry5B under normoxia. In contrast, *hif-1(ia04)* mutant animals on Cry5B PFT look similarly sick either in the normoxic or in the hypoxic environment. Scale bar is 0.2 mm. (C) Schematic illustrating our results and relationship between the HIF-1 pathway and PFT INCED. In response to PFT, the hypoxia response is activated by suppression of EGL-9, either by low oxygen and/or other means. HIF-1 activates expression of target genes that protect against PFT intoxication, such as *nhr-57*. Activation of HIF-1 is also able to activate the XBP-1 arm of the UPR defense pathway.

Although the above data demonstrate the hypoxia pathway is important for PFT INCED, they do not directly address whether the defense against PFTs is related to a low oxygen response or to some other function of the HIF-1 pathway. We therefore examined whether the hypoxia pathway itself is activated by PFTs using *nhr-57* expression, the canonical marker for HIF-1 pathway activation by low oxygen in *C. elegans* (see above). We find that 4 and 8 hours of treatment with PFT significantly induces *nhr-57* expression 5.3 and 3.6 fold respectively (Figure 7A). Shorter treatments with PFT do not. Thus, PFT induces the hypoxia pathway.

If a low oxygen response is involved in responding to PFTs, then one might predict that exposure to low oxygen might confer protection against PFT attack since the low oxygen environment might strongly and rapidly induce the correct protective response. We therefore exposed *C. elegans* hermaphrodites to low (2%) oxygen levels minus or plus the presence of *E. coli*-expressing Cry5B PFT. We find that low oxygen is indeed protective against PFT intoxication in that animals exposed to PFT in a low oxygen environment for 24 hours are significantly healthier than animals exposed to PFT in normoxia (Figure 7B). Similar results were obtained for animals exposed to a low oxygen environment for three days (Figure S4). In contrast and as expected, *hif-1(ia04)* mutant animals exposed to Cry5B PFT do not get any protection when placed in a hypoxic environment (Figure 7B), confirming that the protective effect of hypoxia against PFT is due to activation of the HIF-1 pathway.

## Discussion

Our results demonstrate that the hypoxia pathway protects *C. elegans* against PFTs, whether Bt Cry protein PFTs or a PFT used by a mammalian pathogen, *V. cholerae* VCC. We find that activation of HIF-1 pathway by removal of any of EGL-9/PHD, VHL-1, or RHY-1, makes *C. elegans* more resistant to PFTs than they normally are. This resistance is completely abrogated upon loss of HIF-1, which can additionally result in animals hypersensitive in PFTs. Resistance to PFTs functions in the cells directly targeted by PFTs and is not associated with other hypoxia-mediated stress resistance phenotypes. Furthermore, exposure to PFT induces transcriptional activation of the HIF-1 low oxygen pathway, and exposure of animals to low oxygen protects animals against PFT intoxication, through a HIF-1-dependent mechanism. A schematic summarizing our findings here is in Figure 7C. Consistent with our finding that activation of the HIF-1 pathway is protective against PFTs, it has been shown that expression of the HIF-1α protein is increased in human airway cells by *S. aureus* supernatants, of which α-toxin is a major constituent [44].

The simplest interpretation of our data is that PFT intoxication is associated with low oxygen in cells, and that the hypoxia pathway is therefore needed to protect the cells against this condition. Alternatively, although less parsimoniously, it is possible that both hypoxia and PFTs trigger the same set of HIF-1 downstream mediators that are protective against both assaults but that are not otherwise linked by the presence of low oxygen. Two downstream effectors of the hypoxia PFT INCED pathway are the UPR and *nhr-57*. The fact that *nhr-57* is involved in hypoxia PFT INCED suggests that multiple transcriptional responses are key to mounting an effective defense against PFTs. The link between the XBP-1 UPR, hypoxia, and PFTs is intriguing. It has already been shown in mammalian cells that hypoxia induces activation of the XBP-1 UPR as detected by an up-regulation in *xbp-1* mRNA splicing by low oxygen [45]. Furthermore, it has been shown that XBP-1 protects cells against hypoxia-induced apoptosis [45]. Therefore, we speculate that one role of the hypoxia pathway in PFT INCED is to induce an XBP-1-linked protective response against PFT/low oxygen-mediated apoptosis. Given that the p38 MAPK pathway is also linked to PFT defenses and the UPR, it will be interesting to explore further links between hypoxia, the UPR, p38 and apoptotic pathways in response to PFTs.

Although this report is not the first of hypoxia pathway involvement in immunity, it is the first showing a link between hypoxia and protection of cells that are being directly attacked by a virulence factor. Control of the metabolic shift to glycolysis by HIF-1α has been shown to play an important role in myeloid cell-mediated inflammatory response [46]. Furthermore, it has been shown that bacteria increase HIF-1α protein expression and stimulate HIF-1α transcriptional activity in macrophages, regulating the expression of immune effectors molecules, including antimicrobial peptides, nitric oxide and tumor necrosis factor-α [47]. Our results point to a new and different role of the hypoxia pathway, namely in providing autonomous protection of epithelial cells against PFTs.

To our knowledge, these results are the first to demonstrate that an intracellular pathway can be altered to promote general *resistance* to PFTs. Although a few receptor mutants that confer resistance to PFTs have been previously identified, these do not confer general resistance. A logical extension of our findings is that significant therapeutic benefit against a wide range of bacterial pathogens such as *S. aureus*, *Streptococci*, *Clostrida*, *V. cholerae* (all of which use PFTs as virulence factors) could be achieved by up-regulation of HIF-1 and/or by hypoxia. The identification of the hypoxia pathway as an important PFT INCED pathway thus unexpectedly provides a novel and potentially powerful means of protecting against the single most common mode by which bacterial pathogens attack us.

## Materials and Methods

### *C. elegans* maintenance and microscopy

Strains were maintained at 20°C under standard conditions [48]. The wild-type strain for this study is N2 Bristol [48]. The strains *egl-9(sa307)*, *hif-1(ia04)*, *egl-9(sa307) hif-1(ia04)*, *vhl-1(ok161)*, *rhy-1(ok1402)*, the Hawaiian strain CB4856 and HT1593 [*unc-119 (ed3)*] were obtained from the *Caenorhabditis* Genetic Center (CGC). All strains were either previously outcrossed or outcrossed here at least six times (*e.g.*, *egl-9(ye49)*, *rhy-1(ok1402)*). *egl-9(sa307)* is a null allele of *egl-9* that carries an internal 243-bp deletion removing part of exons 5 and 6 [23]. *hif-1(ia04)* allele removes exons 2, 3 and 4 of *hif-1*, including the DNA binding domain, and is believed to be a null allele [49]. The *vhl-1(ok161)* allele removes exons 1 and 2 of *vhl-1* and is believed to be a null allele [25]. The *rhy-1(ok1402)* allele deletes exons 2, 3 and 4 of *rhy-1* and is also believed to be null [26]. Images were acquired using an Olympus SZ60 dissecting microscope and a Canon PowerShot A620 digital camera.

### Production of a recombinant Cry21A Bt strain and spore-crystal lysates (SCLs)

For production in Bt, the *cry21A* gene was cloned under 700 bp of the *cry6A* promoter region and subcloned into the Bt/*E. coli* shuttle vector pHT3101. The plasmid was transformed into a nontoxic host Bt strain (4D22). Cry21A SCLs were prepared using standard procedures [50] and the concentration was measured relative to BSA standards on protein gels.

### Genetic screen for Cry21A resistance mutants

Mutagenesis and selection of Cry21A resistance mutants was carried out as described for Cry5B [20] except Cry21A SCLs were used to a final concentration of 0.25 µg/mL Cry21A. The 68,000 F2 animals were taken from a larger population of 1,300,000 F2 animals that came from 240,000 mutagenized F1 animals. Animals were incubated for 72 hours at 20°C and scored for overall health, including color, size, movement and brood size. Clonal lines were established from candidates and retested.

### Complementation tests, mapping, and sequencing of *ye49*; rescue experiments

Complementation tests were performed by testing F1 progeny from the cross between *egl-9(sa307)* males and *dpy-17(e164);ye49* hermaphrodites. As a control, cross-progeny from *egl-9(sa307)* males into *dpy-17(e164)* and from N2 males into *dpy-17(e164);ye49* were also tested. *ye49* was mapped between *dpy-11(e224)* and *unc-76(e911)* using standard three-factor mapping. A *dpy-11(e224) ye49 unc-76(e911)* triple mutant was then made in order to perform single nucleotide polymorphism mapping with the Hawaiian strain (CB4856) [51].

Genomic DNA and cDNA prepared from *egl-9(ye49)* animals were used to sequence the *egl-9* gene. For transformation rescue, a 13.4kb-PCR fragment covering from 3kb upstream to 2kb downstream of *egl-9* transcript was amplified with primers GAGCAACTCGTGGGTTTGTT and CTTCCAGAGGCGTTGTCTTC using the LongAmp Taq (Biolabs) from N2 genomic DNA and injected in *egl-9(ye49)* worms as described [52].

For tissue-specific rescue, *egl-9* rescuing plasmids were constructed by PCR amplification of *unc-31* and *cpr-1* promoters and then fused to *egl-9* and *gfp* open reading frames using the Multisite Gateway cloning system (Invitrogen) and pCG150 (containing *unc-119* rescuing fragment) [53]. The constructs were verified by sequencing and integrated into HY0843 [*unc-119(ed3);egl-9(sa307)*] by ballistic bombardment [54] with a PDS-1000/He Biolistic Particle Delivery System (Bio-Rad, Hercules, CA). Two independent lines of each transgenic strain were examined.

### Mortality, morbidity, RNAi and hypoxia assays

For Cry21A *E. coli* toxin assays, we used *E. coli* JM103 with pQE9 empty vector or a *cry21A* gene insert under control of the *lacZ* promoter [11]. Since Cry21A is expressed at very high levels by *E. coli* [11] and too potent for scoring for resistance, we diluted the toxin-expressing bacteria with non-toxin-expressing bacteria at a ratio of 1∶40 for all tests in this study, similar to that previously described for Cry5B studies [13],[15].

Dose-dependent mortality assays with purified Cry5B were performed as described [52]; hermaphrodite viability was scored after 6 days at 25°C. Cry21A SCLs were used for quantitative mortality assays as described above except hermaphrodite viability was scored after 72 hours at 20°C. Each assay was set up with triplicate wells for each concentration of Cry toxin, and each experiment was performed in at least three independent trials. Typically 180 worms were scored for each concentration.

*V. cholerae* lifespan assay was performed as described [55] except the overnight culture was spread on Brain Heart Infusion (BHI) plates. CVD109 *Δ(ctxAB zot ace)* and CVD110 *Δ(ctxAB zot ace) hlyA::(ctxB mer) Hg^r^* strains, derived from *V. cholerae* El Tor E7946, were used [56]. The experiment was performed three times with approximately 50 worms per strain at room temperature (22°C). *P. aeruginosa* lifespan assays were performed on slow-killing plates as described [15]. Heat shock assays were performed as described [57]. For the oxidative stress analysis, synchronized young adults were exposed to 7.5 mM t-butyl hydrogen peroxide as described [58] and were observed after 6 hours. Life-span assays were initiated by allowing adult hermaphrodites to lay eggs on NG plates spread with OP50. When these eggs hatched and the nematodes reached the L4 stage they were transferred to fresh NG plates with OP50 supplemented with 25 µM 5-fluorodeoxyuridine (FUDR) to prevent eggs from hatching. The nematodes were scored for live/dead every 48 hours by tapping the nose at least three times (no movement for all taps was scored as dead).

For RNAi tests, adult hermaphrodites were allowed to lay eggs on NG plates containing 100 µM Isopropyl β-D-1-thiogalactopyranoside (IPTG) and 50 µg/mL ampicillin spread with *E. coli* strain HT115 expressing double-stranded (ds) RNA (from the Ahringer library [59]) for 8 hours and then removed. The eggs were allowed to develop into L4 larvae on RNAi plates at 20°C. L4 hermaphrodites (ten per genotype or line) were picked onto toxin plates spread with 100 µl of a mixture of *E. coli* strain HT115 expressing the same dsRNA and HT115 harboring *cry21A*-expressing vector at the ratio 40∶1. For no toxin control plates, 100 µl of HT115 with dsRNA was spread. *nhr-57(RNAi)* testing on Cry5B PFT was performed slightly differently (Kao *et al.*, manuscript in preparation). Briefly, *rrf-3(pk1426)* animals were fed *E. coli*-expressing dsRNA in liquid media with 1mM IPTG at 25°C for ∼30 h. 20 µg/ml of Cry5B or 20 mM HEPES control were then added, as well as 200 µM FUdR. Hermaphrodites viability was scored after 6 days at 25°C. (As this assay is set up differently, direct comparison with dose-dependent mortality assays presented in Table 1 and associated Figures is not possible).

To test worms under hypoxia, L4 wild type and *hif-1(ia04)* mutant animals were pipetted onto toxin plates spread with 30 µl of a mixture of *E. coli* OP50 strains expressing or not Cry5B at the ratio 1∶1. Plates were placed immediately in a 2% O_2_ chamber for 24 hours, while control plates were placed in room air. Images were taken with an Olympus BX60 microscope as described [15].

### Real time PCR

Real time PCR was performed as described [15]. To determine the levels of spliced *xbp-1* mRNA, we used primers xbp-1_sqf2 GCATGCATCTACCAGAACGTC and xbp-1_sqr2 GTTCCCACTGCTGATTCAAAG to amplify cDNA from wild-type and *egl-9(sa307)* animals. The forward primer xbp-1_sqf2 anneals to exon 1 and the reverse primer xbp-1_sqr2 anneals to the exon1-exon 2 junction sequence produced when intron 1 is spliced out. The experiment was carried out using two independent sets of cDNA and two repeats within each set. Primers TTATCGAGTTTCTCGCATTGG and AAGTCTGCAATCACGCTCTGT were used to quantify expression of *nhr-57*. Induction of expression of *nhr-57* by Cry5B was tested in *glp-4(bn2)* animals treated for 1, 2, 4 and 8 hours on *E. coli* OP50 strains expressing Cry5B or not. The experiment was carried out using three independent sets of cDNA. Normalization in all cases was to *eft-2* transcript levels.

### Statistical analyses

LC_50_ values were determined by PROBIT analysis [60]. Mortality assays were plotted using GraphPad Prism 5.0 (San Diego). Statistical analysis between two values was compared with a paired t-test. Statistical analysis among three or more values of one independent variable was done with matched one-way ANOVA with Tukey's method and of more than two independent variables by two-way ANOVA with the Bonferroni post test. For lifespan analysis, survival fractions were calculated using the Kaplan-Meier method and survival curves compared using the logrank test. Statistical significance was set at P<0.05.

## Supporting Information

Figure S1 *egl-9* mutant animals resist Cry21A PFT-induced sterility. Numbers given are the relative brood sizes of wild-type N2 and *egl-9(ye49)* animals on Cry21A normalized to no-toxin controls (mean of three independent assays). For brood size assays, L4 hermaphrodites from N2 and *egl-9(ye49)* were picked one each to four to six plates and incubated at 20°C. Every 24 h, the originally picked worms would be picked to a new plate; progeny from the old plate were counted 24 h later. This process was continued until the original parents ceased to produce progeny. On *E. coli* plates expressing Cry21A, N2 animals show a 6.2-fold reduction in fertility, while *egl-9(ye49)* show only a 1.5-fold reduction in fertility. Error bar represents standard error of the mean. P = 0.03 (one-tailed T test).
(0.87 MB PDF)

Figure S2 *rhy-1* mutation confers resistance to Cry21A PFT. Dose-dependent mortality assays were performed using Cry21A spore crystal lysates to quantitatively compare sensitivities of wild-type N2 and *rhy-1(ok1402)* mutants. Each data point shows the mean and standard error of the mean from three independent experiments (three wells per experiment; ∼180 animals per data point). Statistical differences between mutant strains and N2 are given for each concentration using P values represented by asterisks as follows: * P<0.05; ** P<0.01; *** P<0.001. LC_50_ values and % alive at specific doses are reported in Table 1.
(1.98 MB PDF)

Figure S3 RNAi confirmation that Cry21A resistance associated with loss of EGL-9 is mediated through HIF-1. Wild-type N2, *egl-9(sa307)*, and *hif-1(ia04)* mutant animals were treated with RNAi of either empty vector (L4440), *egl-9*, *hif-1* or *dpy-3* (positive control for RNAi effectiveness) and exposed to *E. coli* expressed Cry21A PFT for 48 hours. When put on toxin plates, only wild-type animals on *egl-9(RNAi)* and *egl-9(sa307)* on either empty vector, *egl-9(RNAi)*, or *dpy-3(RNAi)* display a resistance phenotype. RNAi of *hif-1* in the presence of *egl-9(sa307)* suppresses Cry21A PFT resistance. RNAi of *egl-9* in the presence of *hif-1(ia04)* does not confer resistance to Cry21A. Scale bar is 0.5 mm.
(4.07 MB PDF)

Figure S4 Hypoxia confers protection against Cry5B PFT. Resistance to Cry5B PFT was compared among wild-type N2 worms in normoxia (top two rows) and in hypoxia (1.5% O_2_) for 72 hours (bottom two rows). Worms co-treated with hypoxia and Cry5B are significantly healthier (larger, darker color, more embryos, more motile) than worms treated with Cry5B under normoxia. Scale bar is 0.2 mm.
(0.55 MB PDF)

Table S1 Data and analyses of VCC, PA14, and lifespan repeat assays.
(0.10 MB PDF)
