# Supplementary material for: Hypoxia and the Hypoxic Response Pathway Protect against Pore-Forming Toxins in C. elegans
Source: PLoS Pathog. 2009 Dec 11;5(12):e1000689. doi: 10.1371/journal.ppat.1000689 (PMC2785477; doi:10.1371/journal.ppat.1000689)
Supplement: Figure S1 — egl-9 mutant animals resist Cry21A PFT-induced sterility. Numbers given are the relative brood sizes of wild-type N2 and egl-9(ye49) animals on Cry21A normalized to no-toxin controls (mean of three independent assays). For brood size assays, L4 hermaphrodites from N2 and egl-9(ye49) were picked one each to four to six plates and incubated at 20°C. Every 24 h, the originally picked worms would be picked to a new plate; progeny from the old plate were counted 24 h later. This process was continued until the original parents ceased to produce progeny. On E. coli plates expressing Cry21A, N2 animals show a 6.2-fold reduction in fertility, while egl-9(ye49) show only a 1.5-fold reduction in fertility. Error bar represents standard error of the mean. P = 0.03 (one-tailed T test). (0.87 MB PDF) [file ppat.1000689.s001.pdf]

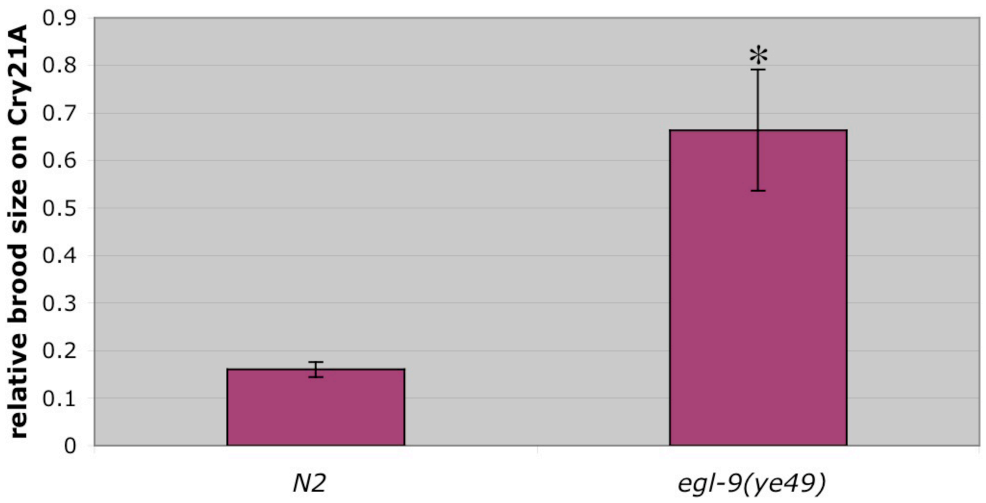

Figure S1. *egl-9* mutant animals resist Cry21A PFT-induced sterility. Numbers given are the relative brood sizes of wild-type N2 and *egl-9(ye49)* animals on Cry21A normalized to no-toxin controls (mean of three independent assays). For brood size assays, L4 hermaphrodites from N2 and *egl-9(ye49)* were picked one each to four to six plates and incubated at 20ºC. Every 24 h, the originally picked worms would be picked to a new plate; progeny from the old plate were counted 24 h later. This process was continued until the original parents ceased to produce progeny. On *E. coli* plates expressing Cry21A, N2 animals show a 6.2-fold reduction in fertility, while *egl-9(ye49)* show only a 1.5-fold reduction in fertility. Error bar represents standard error of the mean. P=0.03 (one-tailed T test).
