# Supplementary material for: Hypoxia and the Hypoxic Response Pathway Protect against Pore-Forming Toxins in C. elegans
Source: PLoS Pathog. 2009 Dec 11;5(12):e1000689. doi: 10.1371/journal.ppat.1000689 (PMC2785477; doi:10.1371/journal.ppat.1000689)
Supplement: Figure S2 — rhy-1 mutation confers resistance to Cry21A PFT. Dose-dependent mortality assays were performed using Cry21A spore crystal lysates to quantitatively compare sensitivities of wild-type N2 and rhy-1(ok1402) mutants. Each data point shows the mean and standard error of the mean from three independent experiments (three wells per experiment; ∼180 animals per data point). Statistical differences between mutant strains and N2 are given for each concentration using P values represented by asterisks as follows: * P<0.05; ** P<0.01; *** P<0.001. LC50 values and % alive at specific doses are reported in Table 1. (1.98 MB PDF) [file ppat.1000689.s002.pdf]

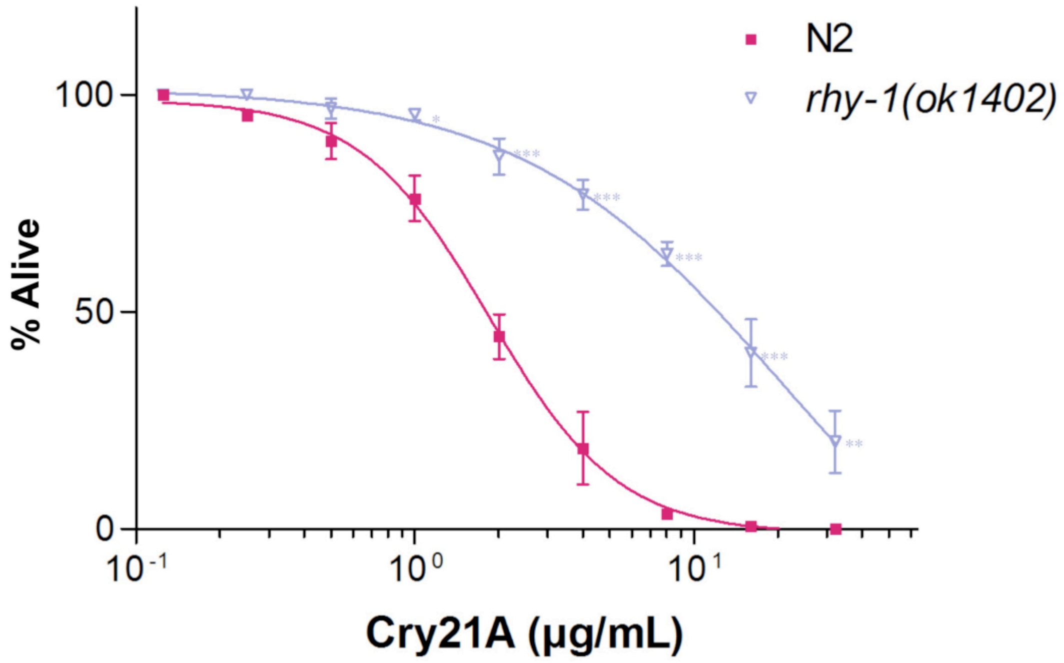

Figure S2. *rhy-1* mutation confers resistance to Cry21A PFT. Dose-dependent mortality assays were performed using Cry21A spore crystal lysates to quantitatively compare sensitivities of wild-type N2 and *rhy-1(ok1402)* mutants. Each data point shows the mean and standard error of the mean from three independent experiments (three wells per experiment; ~180 animals per data point). Statistical differences between mutant strains and N2 are given for each concentration using P values represented by asterisks as follows: * $P<0.05$; ** $P<0.01$; *** $P<0.001$. LC_{50} values and % alive at specific doses are reported in Table 1.
