# Supplementary material for: Hypoxia and the Hypoxic Response Pathway Protect against Pore-Forming Toxins in C. elegans
Source: PLoS Pathog. 2009 Dec 11;5(12):e1000689. doi: 10.1371/journal.ppat.1000689 (PMC2785477; doi:10.1371/journal.ppat.1000689)
Supplement: Figure S3 — RNAi confirmation that Cry21A resistance associated with loss of EGL-9 is mediated through HIF-1. Wild-type N2, egl-9(sa307), and hif-1(ia04) mutant animals were treated with RNAi of either empty vector (L4440), egl-9, hif-1 or dpy-3 (positive control for RNAi effectiveness) and exposed to E. coli expressed Cry21A PFT for 48 hours. When put on toxin plates, only wild-type animals on egl-9(RNAi) and egl-9(sa307) on either empty vector, egl-9(RNAi), or dpy-3(RNAi) display a resistance phenotype. RNAi of hif-1 in the presence of egl-9(sa307) suppresses Cry21A PFT resistance. RNAi of egl-9 in the presence of hif-1(ia04) does not confer resistance to Cry21A. Scale bar is 0.5 mm. (4.07 MB PDF) [file ppat.1000689.s003.pdf]

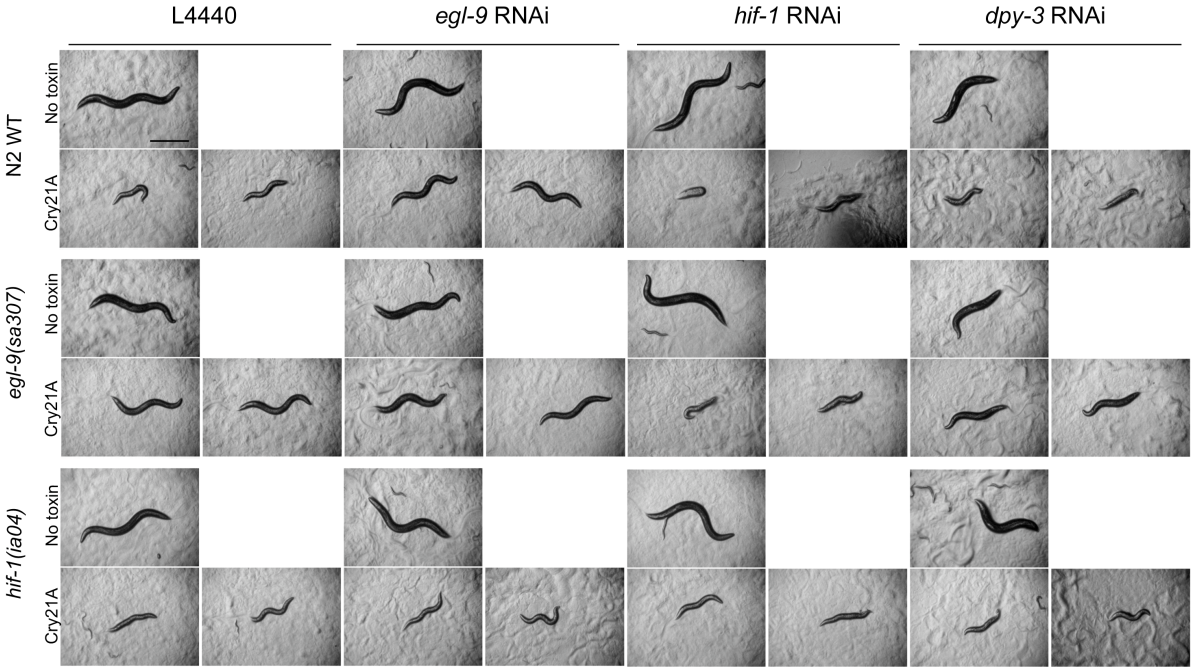

Figure S3. RNAi confirmation that Cry21A resistance associated with loss of EGL-9 is mediated through HIF-1. Wild-type N2, *egl-9(sa307)*, and *hif-1(ia04)* mutant animals were treated with RNAi of either empty vector (L4440), *egl-9, hif-1* or *dpy-3* (positive control for RNAi effectiveness) and exposed to *E. coli* expressed Cry21A PFT for 48 hours. When put on toxin plates, only wild-type animals on *egl-9(RNAi)* and *egl-9(sa307)* on either empty vector, *egl-9(RNAi)*, or *dpy-3(RNAi)* display a resistance phenotype. RNAi of *hif-1* in the presence of *egl-9(sa307)* suppresses Cry21A PFT resistance. RNAi of *egl-9* in the presence of *hif-1(ia04)* does not confer resistance to Cry21A. Scale bar is 0.5 mm.
