# Supplementary material for: Hypoxia and the Hypoxic Response Pathway Protect against Pore-Forming Toxins in C. elegans
Source: PLoS Pathog. 2009 Dec 11;5(12):e1000689. doi: 10.1371/journal.ppat.1000689 (PMC2785477; doi:10.1371/journal.ppat.1000689)
Supplement: Figure S4 — Hypoxia confers protection against Cry5B PFT. Resistance to Cry5B PFT was compared among wild-type N2 worms in normoxia (top two rows) and in hypoxia (1.5% O2) for 72 hours (bottom two rows). Worms co-treated with hypoxia and Cry5B are significantly healthier (larger, darker color, more embryos, more motile) than worms treated with Cry5B under normoxia. Scale bar is 0.2 mm. (0.55 MB PDF) [file ppat.1000689.s004.pdf]

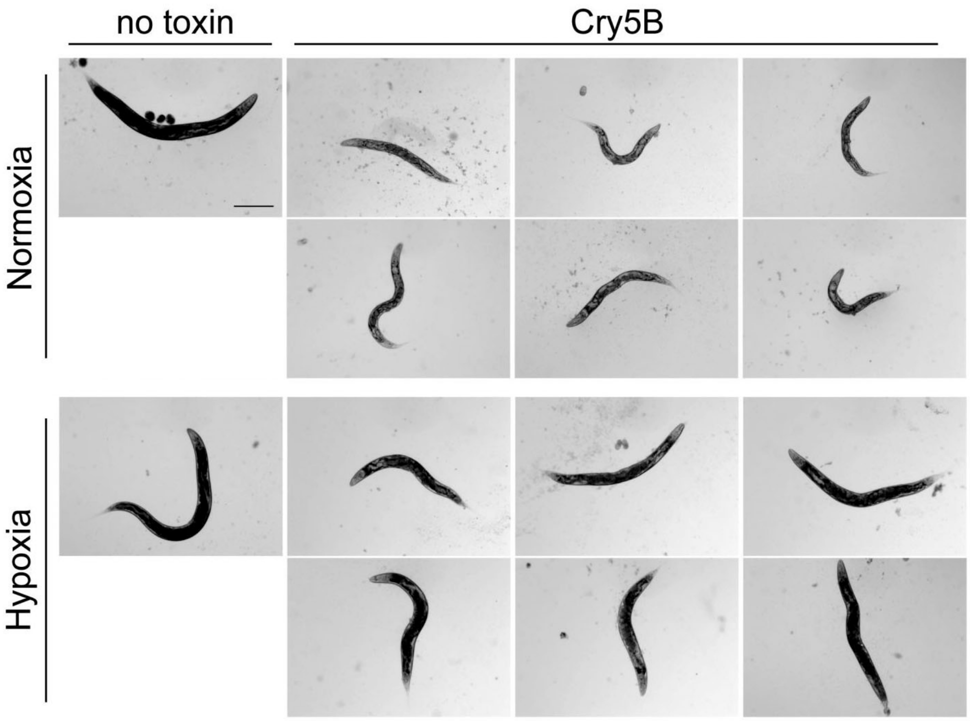

Figure S4. Hypoxia confers protection against Cry5B PFT. Resistance to Cry5B PFT was compared among wild-type N2 worms in normoxia (top two rows) and in hypoxia (1.5% O_2) for 72 hours (bottom two rows). Worms co-treated with hypoxia and Cry5B are significantly healthier (larger, darker color, more embryos, more motile) than worms treated with Cry5B under normoxia. Scale bar is 0.2 mm.
