# Supplementary material for: Hypoxia and the Hypoxic Response Pathway Protect against Pore-Forming Toxins in C. elegans
Source: PLoS Pathog. 2009 Dec 11;5(12):e1000689. doi: 10.1371/journal.ppat.1000689 (PMC2785477; doi:10.1371/journal.ppat.1000689)
Supplement: Table S1 — Data and analyses of VCC, PA14, and lifespan repeat assays. (0.10 MB PDF) [file ppat.1000689.s005.pdf]

Table S1. Data and analyses of VCC, PA14, and lifespan repeat assays

***V. cholerae* assays**	**median survival (days)**	**p value for comparison with the N2 survival curves**
N2/CVD109(VCC+)	2.5	
egl-9(sa307)/CVD109(VCC+)	3	<0.0001
*hif-1(ia04)/*CVD109(VCC+)	1	<0.0001[1]
*egl-9(sa307)hif-1(ia04)/*CVD109(VCC+)	1	<0.0001[1]
N2/CVD110(VCC-)	7	
*egl-9(sa307)/*CVD110(VCC-)	4	<0.0001
*hif-1(ia04)/*CVD110(VCC-)	6	>0.05
*egl-9(sa307)hif-1(ia04)/*CVD110(VCC-)	6	>0.05
N2/CVD109(VCC+)	3	
egl-9(sa307)/CVD109(VCC+)	4	<0.05
*hif-1(ia04)/*CVD109(VCC+)	3	>0.05[1]
*egl-9(sa307hif-1(ia04)/*CVD109(VCC+)	3	>0.05[1]
N2/CVD110(VCC-)	8	
*egl-9(sa307)/*CVD110(VCC-)	5	<0.0001
*hif-1(ia04)/*CVD110(VCC-)	8	>0.05
*egl-9(sa307)hif-1(ia04)/*CVD110(VCC-)	8	>0.05

***P. aeruginosa* PA14 assays**	**median survival (hours)**	**p value for comparison with the N2 survival curves**
N2	55	
egl-9(ye49)	65	<0.0001
egl-9(sa307)	65	<0.001
N2	55	
egl-9(ye49)	70	<0.0001
egl-9(sa307)	65	<0.05
N2	56	
hif-1(ia04)	63	<0.0001
egl-9(sa307)hif-1(ia04)	75	<0.0001
N2	60	
hif-1(ia04)	75	<0.0001
egl-9(sa307)hif-1(ia04)	79	<0.0001

**Lifespan analysis**	**median survival (days)**	**p value for comparison with the N2 survival curves**
N2	15	
egl-9(ye49)	23	<0.01
egl-9(sa307)	21	<0.05
N2	15	
egl-9(ye49)	19	<0.05
egl-9(sa307)	17	<0.05
N2	15	
hif-1(ia04)	19	<0.001
egl-9(sa307)hif-1(ia04)	17	<0.01
N2	14	
hif-1(ia04)	18	<0.01
egl-9(sa307)hif-1(ia04)	18	<0.01

[1]**in the first and second but not the third, *V. cholerae* assays, *hif-1(ia04)* and *egl-9(sa307) hif-1(ia04)* are statistically hypersensitive to CVD109. If all three experiments are combined, both *hif-1(ia04)* and *egl-9(sa307) hif-1(ia04)* are statistically hypersensitive to CVD109 (P<0.0001 for both strains).**
